# Sphingosine-1-Phosphate Receptor Modulators and Oligodendroglial Cells: Beyond Immunomodulation

**DOI:** 10.3390/ijms21207537

**Published:** 2020-10-13

**Authors:** Alessandra Roggeri, Melissa Schepers, Assia Tiane, Ben Rombaut, Lieve van Veggel, Niels Hellings, Jos Prickaerts, Anna Pittaluga, Tim Vanmierlo

**Affiliations:** 1 Department of Pharmacy (DiFar), University of Genoa, Viale Cembrano 4, 16148 Genoa, Italy; roggeri@difar.unige.it; 2 Neuroimmune Connection & Repair Lab, Biomedical Research Institute, Hasselt University, 3500 Hasselt, Belgium; melissa.schepers@uhasselt.be (M.S.); assia.tiane@uhasselt.be (A.T.); ben.rombaut@uhasselt.be (B.R.); lieve.vanveggel@uhasselt.be (L.v.V.); niels.hellings@uhasselt.be (N.H.); 3 Department Psychiatry and Neuropsychology, European Graduate School of Neuroscience, School for Mental Health and Neuroscience, Maastricht University, 6200 MD Maastricht, The Netherlands; jos.prickaerts@maastrichtuniversity.nl; 4 Department of Pharmacy (DiFar), Center of Excellence for Biomedical Research, 3Rs Center, University of Genoa, Viale Cembrano 4, 16148 Genoa, Italy; pittalug@difar.unige.it; 5 IRCCS Ospedale Policlinico San Martino, 16145 Genoa, Italy

**Keywords:** Sphingosine-1-phosphate receptor modulators, OPC, oligodendrocyte, demyelination, multiple sclerosis

## Abstract

Multiple sclerosis (MS) is an autoimmune inflammatory disease characterized by demyelination, axonal loss, and synaptic impairment in the central nervous system (CNS). The available therapies aim to reduce the severity of the pathology during the early inflammatory stages, but they are not effective in the chronic stage of the disease. In this phase, failure in endogenous remyelination is associated with the impairment of oligodendrocytes progenitor cells (OPCs) to migrate and differentiate into mature myelinating oligodendrocytes. Therefore, stimulating differentiation of OPCs into myelinating oligodendrocytes has become one of the main goals of new therapeutic approaches for MS. Different disease-modifying therapies targeting sphingosine-1-phosphate receptors (S1PRs) have been approved or are being developed to treat MS. Besides their immunomodulatory effects, growing evidence suggests that targeting S1PRs modulates mechanisms beyond immunomodulation, such as remyelination. In this context, this review focuses on the current understanding of S1PR modulators and their direct effect on OPCs and oligodendrocytes.

## 1. Introduction

The term neuroglia was coined in the 19th century by Rudolf Virchow and colleagues, who first discovered these extra-neuronal cells in the central nervous system (CNS). The name is derived from the German term “nervekitt,” which literally means nerve glue, since it was initially considered as part of the connective tissue. Although the ratio between neurons and glial cells was under debate for years, it is now assessed to be approximately 1:1 for neuronal and non-neuronal cells, including spatiotemporal differences [1,2,3]. Neuroglia comprises four leading groups of cells: Astrocytes, microglia, oligodendrocytes, and ependymal cells. Oligodendrocytes are the myelinating cells of the CNS and represent about 18% of the total brain cells [4]. They were described first in 1921, by Rio de Hortega, who hypothesized their primary role in myelination [5].

The presence of oligodendrocytes arises during embryonic development and continues after birth. The first pool of oligodendrocyte progenitor cells (OPCs) develops in the ventricular germinal zone around embryonic day 12.5 (E12.5) in mice, E14 in rats, and at gestational week 6.5 (∼E45) in humans [6]. In the spinal cord, oligodendrocytes mainly originate from undifferentiated neuroepithelial cells of the neural tube’s ventral ventricular zone [7]. Oligodendrocytes in the forebrain arise from three distinct waves of OPC differentiation: The first wave starts from the ventral zone, the second from the lateral ganglionic eminence, and the last from dorsal neural precursors [8]. Even though the exact mechanisms are not fully elucidated, it has been elaborately shown that the early differentiation process depends on sonic hedgehog (Shh)-signaling. Shh signaling modulates the expression of transcription factors, such as oligodendrocyte transcription factors 1/2 (Olig1/2), homeobox protein Nkx-6.1 (Nkx6.1), and sex-determining region Y-box 9 (Sox9), which guide gliogenesis [9,10,11,12]. Due to different mitogenic stimuli, mainly mediated by platelet-derived growth factor-alpha (PDGFα) [13,14], OPCs migrate from the site of origin through the vascular system and widely colonize the grey and the white matter in the CNS [15,16] before starting to differentiate into mature myelinating oligodendrocytes. However, a pool of undifferentiated OPCs remains in the parenchyma of the adult CNS. Undifferentiated OPCs are characterized by the expression of chondroitin sulfate proteoglycan NG2, and for this reason, are referred to as NG2^+^ cells. They represent 5% of the total progenitor cells in the adult CNS [17]. They are equally distributed in all the CNS regions, with a numerical density of about 1.5 times higher in the white matter compared to gray matter [17]. NG2^+^ cells are usually considered to be OPCs or even pre-OPCs because they express antigens shared with the oligodendrocytes lineage, and they can give rise to oligodendrocytes both in vitro and in vivo [18,19]. Interestingly, in mice, postnatal forebrain, only half of the NG2^+^ cells are mitotically active lifelong, while the other half is quiescent. The active subpopulation continuously differentiates into oligodendrocytes, but the rate of production declines with age in parallel with a reduction in the proliferative rate of NG2^+^ cells [20].

### 1.1. Oligodendrocyte Differentiation

Both during development and adulthood, OPCs (or NG2^+^ cells) are induced to differentiate following environmental stimuli. Differentiation of OPCs into mature oligodendrocytes includes intermediate states, such as pre-oligodendrocytes, differentiated oligodendrocytes, mature oligodendrocytes, premyelinating, and finally, myelinating oligodendrocytes. The stages are characterized by different cellular morphologies, switching from the bipolar shape of OPCs to the multi-branched mature cells [21]. Furthermore, distinct protein expression patterns arise during differentiation, leading to stage-specific oligodendrocyte markers. OPCs express the membrane antigen A2B5, the chondroitin sulfate proteoglycan NG2, and the PDGFα receptor (PDGFRα) [22]. Throughout the transition to pre-oligodendrocytes, OPCs lose the progenitor markers and start to express other cell surface markers, such as O4, O1, and protein proteolipid protein (PLP) [22]. Pre-oligodendrocytes can further differentiate into myelinating, mature oligodendrocytes, and express the associated myelin proteins, such as myelin basic protein (MBP), myelin-associated glycoprotein (MAG), the membrane marker galactocerebroside (GalC), and surface marker myelin-oligodendrocyte glycoprotein (MOG) [23]. All the steps are accurately tuned by extrinsic and intrinsic factors, determining the correct timing of OPC proliferation, migration, and differentiation. Stimuli, including a diversity of extracellular signals, growth factors, transcription factors, and epigenetic modulators, play a key role in the process of myelin formation [24,25,26].

### 1.2. Oligodendrocytes and Remyelination

Myelin is a fat-enriched plasma membrane extension of oligodendrocytes that wraps the neuronal axons, mediates electrical pulse propagation, and provides nutritional support to axons. Myelin plasticity entails physiological oligodendrocytes turnover, activity-induced deposition of new internodes, and variations in internode length along axons [27]. Alterations in myelination occur throughout the entire lifespan and are essential for acquiring cognitive abilities, such as motor skill learning and memory consolidation [28,29,30,31]. The physiological turnover of myelin in the adult CNS is assured by NG2^+^ cells. The progenitors ensure efficient oligodendrocyte production and maintain the physiological level of myelination [32].

Demyelination describes the pathological loss of myelin, resulting in axon dysfunction, and eventually, axonal loss. Acquired demyelinating diseases can be classified according to the cause of demyelination: Inflammatory processes, viral infections, acquired metabolic derangements, and hypoxic-ischemic-dependent or focal compression [33]. Multiple sclerosis (MS) belongs to the first group and is the most prevalent form of pathological demyelinating disorders in the CNS. Although the etiology remains unknown, MS is usually defined as an autoimmune disease characterized by autoreactive lymphocytes directed against myelin proteins, such as MBP, PLP, and MOG [34]. Another essential hallmark of the disease is the loss of blood-brain barrier (BBB) integrity. Alterations of tight junction proteins [35,36,37] and the consequent increased BBB permeability, ease the passage of autoreactive T-cells towards the CNS [35,38]. Once in the CNS, T-cells start to produce inflammatory mediators leading to demyelination and axonal loss. However, the CNS possesses a physiological repair mechanism, i.e., remyelination, mediated via OPC migration, proliferation, and differentiation. Despite active remyelination during the early stages of MS, remyelination becomes gradually insufficient, ushering the transition to chronic progressive stages of MS. In spite of the recently proven contribution of mature oligodendrocytes to the remyelination process [39], it is mostly recognized that the deficit in OPC migration to the site of injury [40,41] and the block of OPC differentiation [42,43] play a pivotal role in the failing of CNS repair. The exact reason why remyelination is hampered during MS is still under debate. However, the decreased responsiveness of OPCs to external stimuli, as well as the overexpression of inhibitory factors, seem to be the leading causes [44,45,46].

Additionally, OPCs show high vulnerability to oxidative stress that occurs due to the overall increased inflammatory state [47]. OPCs exhibit a remarkably low antioxidant capacity compared to other cell types, as well as higher production of reactive oxygen species (ROS) [48,49,50]. Oxidative stress can be linked to the OPC differentiation block, since ROS are capable of compromising a variety of genetic, epigenetic, and metabolic mechanisms usually crucial for differentiation [26,47,51].

Nowadays, all the disease-modifying therapies (DMTs) available for MS aim to suppress the inflammatory signaling during early disease stages. Currently, preclinical [52,53,54,55,56] and clinical (see. Table 1) studies focus on drugs that can directly target oligodendrocytes or their precursors to enable or boost remyelination. Identification of factors that relieve the block in OPC differentiation will allow restoring endogenous remyelination, holding potential as a strategy predicted to improve disability and limit disease progression significantly.

In this review, we will focus on sphingosine-1-phosphate receptors (S1PRs) as targets to treat MS. In particular, the role of S1PRs modulation in OPC differentiation will be discussed.

## 2. Sphingosine-1-Phosphate Receptors Expression

**Figure 1:**
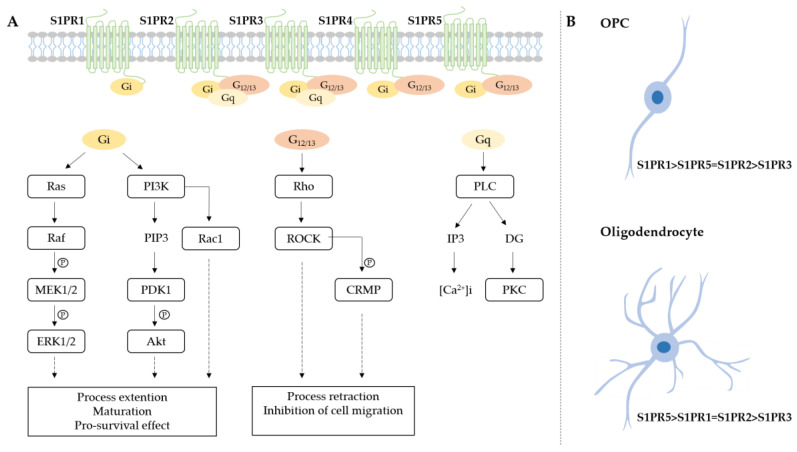
Sphingosine-1-phosphate receptors (S1PRs) expression and main transduction pathways activated in oligodendrocytes progenitor cells (OPCs) and oligodendrocytes. (**A**) Schematic representation of G-protein coupled receptors and main pathways involved after receptor activation. Sphingosine-1-phosphate receptor 1,2,3,5 (S1PR1,2,3,5); mitogen-activated protein kinase (MEK); extracellular signal-regulated kinases (ERK); phosphatidylinositol 3-kinase (PI3K); phosphatidylinositol (3,4,5)-trisphosphate (PIP3); 3-phosphoinositide dependent protein kinase-1 (PDK1); protein kinase B (Akt); phospholipase C (PLC); protein kinase C (PKC); Rho-associated protein kinase 1 (ROCK); collapsin response mediator protein (CRMP). (**B**) S1PRs expression levels in human OPCs and oligodendrocytes [71,72,73,74,75].

Sphingosine 1-phosphate (S1P) is a bioactive sphingolipid metabolite involved in a wide range of cellular and physiological processes, including immune responses and neuronal development [64,65]. S1P is produced through the phosphorylation of sphingosine by one of the two sphingosine kinases, SphK1, and SphK2 [66]. Besides its intracellular effector mechanisms, S1P is usually transported out of the cells, where it can activate intracellular signaling cascades through its binding to S1P G-protein-coupled receptors (S1PRs), in an autocrine or paracrine manner [67,68]. Five known S1PRs (S1PR1, S1PR2, S1PR3, S1PR4, S1PR5) have been described, each with their cellular distribution pattern and differential coupling to diverse G proteins [67,69]. For instance, S1PR1 exclusively activates members of the Gi family, whereas S1PR2 and S1PR3 have a broader coupling profile and also activate Gq and Gα_12/13_ (Figure 1A). The subsequent activation of small GTPases, such as Rho, Rac, and Ras, results in a downstream signaling cascade through distinct second messengers, including mitogen-activated protein (MAP) kinases, phosphoinositide 3-kinase (PI3K), phospholipase C (PLC), and intracellular calcium stores [67,70]. S1P can, hence, influence many cellular processes, such as proliferation, survival, migration, and differentiation, depending on the expression and activation of specific S1PRs [65].

Even though S1PRs are widely expressed in almost every tissue, their subtype-dependent distribution pattern facilitates their functional specificity. One of the major described roles of S1PRs is their involvement in immune cell trafficking [76]. The S1P/S1PR1 binding, in particular, has been shown to modulate lymphocyte migration, as loss of S1PR1 prevents lymphocytes from exiting the lymphoid organs and entering the bloodstream [66,77]. Within the innate immune system, natural killer (NK) cells express virtually all S1PRs, yet S1PR5 expression, in particular, seems to be crucial for NK cells to egress from the lymph nodes and bone marrow [78]. In addition to cellular migration, S1PRs have been shown to be involved in other immunological processes, such as cell-fate switching, cell survival, and antitumor immune responses [79].

S1PRs are also widely expressed within the CNS, and show a cell-specific distribution pattern [65,80]. For instance, the endothelial cells of the BBB express S1PR1, S1PR3, and S1PR5, which contribute to maintain BBB integrity and prevent vascular permeability [81,82]. Both astrocytes and microglia express mainly S1PR1 and S1PR3, which seem to be upregulated during the reactive state of the cells and neuroinflammation [80]. Neuronal precursor cells predominantly function through S1PR1 expression to stimulate neuronal development and neurite outgrowth, yet neurons express S1PR2 and S1PR3 receptors [83]. Interestingly, S1PR5 expression is exclusively shown by oligodendrocytes within the CNS parenchyma [76,80]. As these cells represent the main focus of this review, we will discuss the involvement of S1PRs during oligodendroglial development, OPC differentiation, and myelination in detail.

### Sphingosine-1-Phosphate Receptor Signaling in Oligodendrolineage Cells

Over recent years, considerable evidence shows that activating S1PRs modulates OPC and oligodendrocyte signaling. Only four out of five S1PRs are found in oligodendrolineage cells (S1PR1, S1PR2, S1PR3, S1PR5), and their expression pattern differs during the maturation stages. In OPCs, S1PRs expression is differentially modulated by PDGF, a growth factor primarily produced by proliferating OPCs [84]. OPCs display a preferential expression of S1PR1 and lower levels of S1PR2, S1PR3, and S1PR5 [71,72] (Figure 1B). During differentiation, S1PR1 expression significantly lowers, while S1PR5 becomes the dominant subtype [71,72,73,74,75,84,85,86]. These tightly controlled changes in S1PR subtype expression emphasize the potential of exploiting S1PR signaling for modulating distinct OPC and oligodendrocyte-specific processes.

Stimulating S1PR signaling in both rodent and human oligodendrocytes promotes the cell’s survival [73,84,85]. The protective activity of S1PRs in oligodendrocytes is absent in OPCs. The downstream analysis has shown that S1PR signaling activates and phosphorylates extracellular signal-regulated kinases ½ (ERK1/2) and protein kinase B (Akt) specifically in oligodendrocytes and not OPCs, which subsequently protects them from stress-induced apoptosis [73,84,85]. Interestingly, in human mature oligodendrocytes, the cytoprotective feature of full S1PRs was activation mimicked using an S1PR5 subtype-specific agonist, raising the possibility of attributing the effect of S1PR agonists to specific receptor subtypes [73].

Furthermore, silencing S1PR1 attenuated the PDGF-induced proliferation of OPCs, showing that S1PR1 and S1PR5 serve different functions during oligodendroglial development [84].

In vitro studies have shown that S1PRs activation by low nanomolar concentrations of either S1P or fingolimod—a broad-spectrum agonist—results in enhanced OPC differentiation into both pre-oligodendrocytes and mature oligodendrocytes. Treatment with the S1PRs agonist mediates phosphorylation of ERK1/2, cAMP response element-binding protein (CREB), and p38 mitogen-activated protein kinase (p38MAPK) in bipolar O4^+^ cells [87]. However, in more differentiated O4^+^ cells, only p38MAPK was phosphorylated following treatment with fingolimod [87]. Pre-treatment with mitogen-activated protein kinase (MEK) and p38MAPK inhibitors reduce the agonist-mediated increase in O4^+^ cells, but only p38MAPK inhibition results in a decrease in the differentiation of O4^+^ cells into the mature stage [87]. The results demonstrate that the ERK1/2 pathway regulates the maturation of OPCs into pre-oligodendrocytes, but not the transition to mature oligodendrocytes, that is mediated by p38MAPK signaling.

Interestingly, other studies have shown that activation of S1PR5 inhibits differentiation of OPCs, while activation of S1PR1 enhances the differentiation process [75]. Consistently, animals lacking the S1PR5 subtype develop a regular myelination pattern, while S1PR1 null mice show decreased levels of myelin proteins, thinner myelin sheaths, and increased vulnerability to chemically induced demyelination, highlighting a more prominent involvement of the S1PR1s in the context of myelination [74,88]. Along similar lines, S1PR1 deletion in the oligodendroglial lineage of mice leads to a delay in OPC differentiation into mature oligodendrocytes during early myelination [89].

It is crucial to recognize that physiological and pathological (re)myelination are distinct processes that are regulated differentially and may require different S1PR subtype activation. As is also the case with OPC differentiation, the primary subtype involved in the potential remyelinating features of full S1PR agonists remains mostly unknown. However, it appears plausible that S1PR1, mainly expressed in OPCs, can play a pivotal role in early differentiation. At the same time, S1PR5, the most representative subtype in the oligodendrocyte, may be involved in late myelination processes.

Although the S1PR5 subtype is expressed at lower levels in OPCs compared to oligodendrocytes, and no survival-promoting features were observed when stimulating this receptor subtype in OPCs, S1PR5 activation by S1P blocks OPC migration in a transwell migration assay [71]. The impaired migratory capacity upon S1PR5 activation is thought to be explained by the engagement of Gα_12/13_ protein. The activation of these proteins is coupled to the Rho/Rho-associated protein kinase 1 (ROCK) signaling pathway, which negatively affects the cellular migratory responses [71,90] (Figure 1A). Consequently, the impeded OPC migration was prevented when knocking down solely S1PR5 [71]. These data are also consistent with the physiological effect of S1PR5 activation in mouse OPCs. In vitro, S1P induces a dose-dependent retraction of the cellular processes in the pre-oligodendrocytes, but it is devoid of activity in mature oligodendrocytes. The retraction is transient, and it is mediated by the activation of S1PR5 receptors and the consequent Rho kinase-mediated phosphorylation of collapsin response mediator protein (CRMP; Figure 1A) [74]. It is interesting to notice that S1P-mediated activation of S1PR5 in mature oligodendrocytes displays, instead, a pro-survival activity. Curiously, also, in this case, the effect is mediated by the S1PR5 activation, but through a pertussis toxin-sensitive Gα/i-protein, which correlates with the activation of Akt signaling [74]. These results underscore that different functions of S1PR5 activation during differentiation correlate to changes in receptor coupling with heterotrimeric G-proteins, thus leading to the activation of different signaling pathways.

Taken together, it is becoming more recognized that the dose and timing of S1PR activation are crucial to regulating distinct OPC and oligodendrocyte related processes. It has been shown that S1PR signaling activation promotes oligodendrocyte survival, migration, differentiation, and myelination, although contradictory results have been observed. S1PR subtype-specific features can potentially explain the variety of biological oligodendrolineage related processes. For instance, activating S1PR1 specifically increases the proliferative rate, while activating S1PR5 specifically regulates OPC migration and oligodendrocyte survival. However, both S1PR1 and S1PR5 may contribute to the observed increased differentiation and myelination properties of S1PRs agonists, although their exact role remains inconclusive so far.

## 3. Sphingosine-1-Phosphate Receptors and Multiple Sclerosis Therapy

In the last decade, many DMTs that target S1PRs have been developed to treat MS. These modulators are structurally similar to the endogenous S1P and are S1PRs agonists. However, their mechanism of action is defined as “functional antagonist”, since the binding to S1PRs causes receptors internalization, ubiquitylation, and subsequently degradation [91]. This mechanism has been recognized as the main reason behind the immunomodulatory effect of the molecules. As the S1P/S1PR1 axis is known to be crucial for lymphocytes migration from the secondary lymphoid nodes, S1PR1 internalization leads to a general lymphopenia, which, in turn, reduces the T-cells infiltration in the CNS [92]. Starting from this evidence, the inhibition of the S1PR1-mediated signaling pathway in the presence of functional antagonists is expected. However, a study conducted in 2009 demonstrated that S1PR1 internalization triggered by fingolimod—an S1PR functional antagonist—results in persistent downstream activation of the S1P-dependent signaling pathway [93]. In the study, short incubation (1 h) of Chinese hamster ovary with 1 µM fingolimod lead to a long-lasting activation of Gi and increased ERK phosphorylation [93]. Another hypothesis suggested an intracellular role of internalized S1PR1 [94]. In S1P treated endothelial cells, internalized S1PR1 translocates to the nuclear compartment and regulates the transcription of growth factors [94]. Consistently, similar re-localization was observed in activated T-cells, where S1PR1 nuclear translocation results in shifting from a pro-migratory to a proliferative response [94]. Although not thoroughly investigated, this mechanism adds new valuable knowledge about the potential effects of S1PR modulators. Indeed, besides the well-establish immunomodulatory effect, S1PR modulators are now investigated for their activity on CNS cells. Many studies are now focusing on the impact of these compounds on OPCs and oligodendrocytes to investigate their possible role in demyelination/remyelination processes.

### 3.1. Sphingosine-1-Phosphate Receptor Modulators Approved for Multiple Sclerosis Treatment

#### 3.1.1. Fingolimod

Out of the many available S1PR modulators, three have been approved by the US food and drug administration (FDA) to treat different forms of MS (Table 2). The first-in-class of these molecules is fingolimod, approved in 2010 as the first oral treatment for the relapsing-remitting form of MS (RRMS). Both in humans and rats, the pro-drug fingolimod is phosphorylated in vivo by SphK2 [95,96] to form the bioactive fingolimod-P. Due to the chemical structure similarity with S1P, fingolimod-P binds four of the five S1P receptors, showing a high affinity to the receptor subtypes 1, 3, and 5, a low affinity to S1PR4, and no activity towards S1PR2 [97]. In experimental autoimmune encephalomyelitis (EAE) rats, fingolimod rapidly crosses the BBB and is phosphorylated by CNS cells. The brain concentration of fingolimod-P not only remains stable, but increases over time [98]. The capability of fingolimod to reach high concentrations in the CNS makes studying its potential activity directed to oligodendroglial cells intriguing.

Thus, it has been shown that in the CNS, fingolimod stimulates the production of neurotrophic factors, such as brain-derived neurotrophic factor (BDNF), leukemia inhibitory factor (LIF), and heparin-binding EGF-like growth factor (HBEGF) from neurons and astrocytes [107,108]. This upregulation can, indirectly, exert a positive effect on OPC and oligodendrocyte differentiation, maturation, and survival, which are known to be influenced by neurotrophic factors [109,110,111].

A vast number of studies have established that fingolimod has a direct action on oligodendrolineage cells, depending on their differentiation stage. In vitro studies on rat OPCs demonstrate that fingolimod treatment exerts a cytoprotective role, reducing cell apoptosis by activating PI3K/Akt and MEK/ERK1/2 signaling pathways [85]. Consistently, in vitro administration of nanomolar concentrations of fingolimod increases the MBP protein levels, and consequently, the percentage of mature oligodendrocytes, whereas micromolar concentrations counteract this positive effect and even inhibited differentiation [84,112,113]. In line with the differentiation promoting features of S1PRs agonists, ex vivo studies also showed an enhanced remyelination capacity when low concentrations of fingolimod (0.1 nM) were administered to either lysolecithin (LPC)-induced demyelinated cerebellar brain slices or rat CNS spheroid cultures [114,115]. Interestingly, the remyelination feature of S1PRs signaling is thought to be primarily mediated by S1PR3 and S1PR5, while ruling out S1PR1 activation [115]. Even if the mechanism remains unclear, high concentrations of fingolimod may lead to the activation of all the receptor subtypes and the loss of receptor-specific activity. In line with this evidence, the activity of fingolimod is intimately dependent on the receptor expression pattern of oligodendrolineage cells. Interestingly, fingolimod itself seems to influence S1PR transcription in mature human oligodendrocyte. A time-dependent regulation of S1PRs expression has been observed following prolonged treatment with high fingolimod concentrations (1 μM). Transcription of S1PR5 mRNA appears to be downregulated at the beginning of the treatment, while after two days, its expression is significantly increased, to then be again cut down at day 8 [73]. Interestingly, at the same time points, S1PR1 mRNA appears to be oppositely regulated, while S1PR3 and S1PR4 expression is not affected [73]. These results suggest that S1PR5 and S1PR1, which mediate different downstream pathways, change their expression level in mature oligodendrocytes, leading to a different functional response to fingolimod. It could be argued that the S1PRs internalization induced by low doses of fingolimod leads to the activation of a compensatory pathway, which in turn enhances the transcription of other S1PRs. This mechanism may result in a continuative activation of S1P-related pathways.

Both in vitro and in vivo research has been conducted regarding S1PRs signaling in different animal models for the study of MS (e.g., EAE model, cuprizone model). Although contradictory results have been obtained regarding (re)myelination and S1PRs signaling, an increase in vivo OPC proliferation, differentiation, and remyelination upon full S1PR activation was observed. Some research groups found an increased number of mature oligodendrocytes in the proximity of demyelinated areas upon fingolimod treatment in the cuprizone model, indicating a potential effect on differentiation or migration without apparent effects on remyelination [116,117,118]. In contrast, others observed accelerated remyelination upon S1PRs activation following fingolimod treatment (0.3 mg/kg) in the acute cuprizone-induced demyelination model, leaving the exact role of S1PRs activators in the cuprizone model inconclusive [119]. Until now, the reason for these discrepancies has not been clearly investigated. Nevertheless, the use of different dosages of fingolimod—1 mg/kg vs. 0.3 mg/kg—as well as the timing of administration, can play a crucial role. Interestingly, a study conducted in 2018, demonstrated that daily intraperitoneal injections of fingolimod (1 mg/kg) three days after the start of cuprizone intoxication prevents mature oligodendrocyte death in the corpus callosum [120]. Differently, the same administration started ten days after cuprizone intoxication does not possess anti-apoptotic properties [120]. The outcomes lead to the conclusion than early intervention is required to prevent demyelination.

Unlike other preclinical models and human disease, cuprizone causes demyelination directly damaging oligodendrocytes, bypassing the autoimmune component. Even if the mechanism of toxicity is not fully elucidated, mitochondria impairment and oxidative stress could play a key role [121]. Studies have shown that several stressors (e.g., hydrogen peroxide, tert-butyl hydroperoxide) severely impair OPC differentiation, and therefore, hamper remyelination processes [47,122]. The exact mechanisms behind this remain unclear; however, several essential pathways involved in DNA damage and repair, the epigenetic machinery and metabolic processes might be involved. The higher occurrence of oxidative DNA damage and impaired repair compared to other CNS cell types, challenge the genomic stability of OPCs [51,123,124]. Furthermore, ROS can negatively impact DNA methylation, histone acetylation, and miRNA presence, which are all deemed necessary in OPC differentiation and especially relieving its blocking mechanisms [26,125,126,127,128]. Finally, the interplay between the metabolic functioning of mitochondria and ROS has been quite well established [129]. Unsurprisingly this relationship is present in OPCs, which heavily rely on mitochondrial ATP production, due to their high energy requirement [130]. All in all, the relationship between OPCs and oxidative stress with regards to the observed differentiation block in MS is an attractive therapeutic target to explore.

Taking all of this into consideration, the positive outcomes of fingolimod in cuprizone-induced demyelination can lead to propose a new mechanism of action. A recent in vitro study on mouse neuronal cells demonstrated that the oxidative damage induced by menadione is counteracted by the incubation with fingolimod (50 nM) [131]. The compound decreased the level of ROS and enhanced the expression and activity of protective factors, such as nuclear factor erythroid 2–related factor 2 (Nrf2), heme oxygenase-1(HO-1), and NAD(P)H: Quinone acceptor oxidoreductases-1 (NQO1) [131]. Consistently, increased expression of Nrf2 was observed in human iPSC-derived astrocytes treated with 100 nM of S1PR modulators, fingolimod, and siponimod [132]. More recently, increased oligodendrocyte loss and demyelination have been observed in the cuprizone-induced demyelination in Nrf2-knockout mice [133]. Since high ROS production by oligodendrocytes mitochondria is involved in demyelination processes, the antioxidant activity of fingolimod and other S1PRs could be a possible explanation of their direct effect on oligodendroglial cells.

In comparison to the cuprizone model, the role of S1PR agonists on OPC modulation in the chronic EAE model has been less investigated. Recently, it has been shown that fingolimod ameliorates remyelination in the EAE model through enhanced OPC proliferation and differentiation. EAE mice were administered fingolimod (0.3 mg/Kg) daily, through an oral gavage, starting from the day of EAE onset. At day 7 post-onset (p.o.), no differences in MBP level could be detected between control and fingolimod treated EAE mice, indicating that a similar demyelination level occurred in both groups at the early stage of the disease. However, fingolimod treatment significantly increased MBP expression at day 30 p.o. and led to an increase in myelinated areas in the lumbar levels of the spinal cord [134]. At the same time, fingolimod administration enhanced the number of proliferating NG2^+^ and induced OPC differentiation, as demonstrated by the increased number of BrdU^+^-CNPase^+^ cells in the striatum, corpus callosum, and spinal cord of EAE mice [134].

Even in the LPC-induced demyelination rat model, fingolimod increased the number of OPCs after six days post-LPC administration. Consistent with the in vitro results, the effect is strictly related to low doses of fingolimod (0.3 mg/Kg), while a high dosage prevented this activity [135]. Therefore, the dosage of administration appears to be crucial to achieving the beneficial effect on OPC and myelination.

While the data from in vitro experiments are more consistent, the in vivo outcomes appear to be more controversial. Different models, dosages, timing of treatment, and analysis of the results could contribute to generate contradicting conclusions. Yet, the positive outcomes obtained on fingolimod treatment in OPCs and oligodendrocytes and the reduction of brain volume loss registered in RRMS patients—FREEDOM (FTY720 Research Evaluating Effects of Daily Oral therapy in Multiple Sclerosis) II clinical trial [136]—suggested that the fingolimod could be used not only for RRMS treatments, but also in other forms of MS. The beneficial effect of fingolimod was assessed to treat the primary progressive form of MS (PPMS), for which only one compound—the monoclonal antibody ocrelizumab—has been approved by the FDA/EMA [137,138]. Despite the promising results obtained in the RRMS, fingolimod failed to significantly slow disease progression in patients with PPMS [139].

Two clinical trials, FREEDOMS and TRANSFORMS (TRial Assessing injectable InterferoN versus FTY720 Oral in RRMS), demonstrated the efficacy and safety of fingolimod in RRMS patients [136,140]. Even though fingolimod is considered safe, clinical trials unveiled a transient and dose-dependent decrease in heart rate (bradycardia) and atrioventricular block [136,141] mainly attributed to the activation of the S1PR3 receptor subtype [142].

In this context, many other S1PR modulators have been developed to treat MS, aimed to produce compounds that selectively target only S1PR1 and S1PR5, the main effectors of fingolimod activity on immune and CNS cells. In addition, ligands that do not bind the S1PR3 could be beneficial for reducing fingolimod-related cardiac side effects.

#### 3.1.2. Siponimod

Siponimod is a second-generation modulator of S1PRs, approved in 2019 by the FDA to treat clinically isolated syndrome (CIS), RRMS, and active secondary progressive (SP) MS. In contrast to fingolimod, siponimod does not require phosphorylation and binds with nanomolar affinity S1PR1 and S1PR5, displaying low affinity toward the other receptors [143] (Table 2). The phase III clinical trial EXPAND (EXploring the efficacy and safety of siponimod in PAtients with secoNDary progressive multiple sclerosis) investigates the effect of siponimod vs. placebo in SPMS patients. While the safety profile was similar to fingolimod—bradycardia and bradyarrhythmia at first-dose treatment—some positive outcomes have been obtained in SPMS patients [144]. Despite the short period of time taken into consideration for the clinical trial, the treatment was approved for the active SPMS. However, further investigation on long-term effects should be performed.

Like its predecessor, siponimod carries out the immunomodulatory effect mainly by reducing the number of circulating lymphocytes [143,145]. However, some evidence suggests its direct involvement in the control of CNS activities. In a modified EAE model with cytokines-mediated induction of cortical grey matter and subcortical white matter lesions, systemic, but not intracerebral administration of siponimod (3 mg/kg) ameliorated the clinical symptoms of EAE mice [146]. Similarly, in the EAE mouse model, continuous intracerebroventricular infusion of siponimod (0.45 μg/day) exerted a positive effect on the imbalanced synaptic transmission that occurs during the disease. Both in vivo and in vitro, siponimod enhanced the inhibitory tone increasing GABA transmission by promoting GABAergic interneuron survival [147]. Physiologically, the glutamate homeostasis at the synaptic cleft is also maintained by the astrocytic glutamate-aspartate transporter (GLAST) and glutamate transporter-1 (GLT-1). Their role is to uptake the glutamate from the synaptic cleft and keep its extracellular concentration below neurotoxic levels. However, a high level of inflammatory mediators leads to decreased receptors’ expression resulting in an increased glutamate amount. In this context, it has been recently observed that, in human iPSC-induced astrocytes, the treatment with high concentrations of siponimod (100 nM) maintains the expression of GLAST and GLT-1 [132], contributing to restoring the imbalanced glutamate transmission.

Although the potential effect of siponimod in the CNS [148], only limited data are available on its direct effect on the oligodendroglial lineage. Studies conducted in the last few years show that siponimod attenuated and prevented demyelination in animal models of MS. Ex vivo, siponimod activity was assessed using LPC-treated rat organotypic cerebellar slice cultures. The slices were exposed to LPC (0.5 mg/mL) in the presence of siponimod (10 nM) for 18 h and treated for a further 30 h with siponimod at the same concentration. The results show that siponimod attenuated the LPC-induced demyelination, and contemporary decreased interleukin (IL)-6 expression [149]. Moreover, daily oral administration of siponimod (0.11 mg/kg) during cuprizone-induced demyelination in mice (4.5 week cuprizone + rapamycin) reduced the axonal damage and decreased the loss of both myelin proteins and mature oligodendrocytes [150]. Differently, siponimod administration during the remyelinating phase (4.5-week cuprizone + rapamycin followed by three weeks of regular diet) lacked these positive effects [150]. All these preliminary data lead to the conclusion that siponimod may intervene in reducing demyelination and promoting the oligodendrocytes’ survival. However, other studies are required to assess the direct impact on OPC/oligodendrocytes proliferation, differentiation, migration, and survival.

#### 3.1.3. Ozanimod

Ozanimod is the most recent S1PR modulator approved in March 2020 by the FDA to treat CIS, RRMS, and active SPMS [151]. Similar to siponimod, it is an active agonist of S1PR1 and S1PR5, and it displays a significantly lower affinity for S1PR2, S1PR3, and S1PR4 [101]. It has a shorter half-life (19 h; Table 2) than the other two compounds, and no first-dose clinically significant bradycardia or atrioventricular block has been reported [152,153]. SUNBEAM (safety and efficacy of ozanimod versus interferon beta-1a in relapsing multiple sclerosis) phase III clinical trial confirm the tolerability of ozanimod at both the doses used—1.0 mg and 0.5 mg—. Despite the same safety profile, only the higher dose was able to reduce the annualized relapse rate (ARR) and the number of new or enlarging T2 and gadolinium-enhancing lesion [153]. In addition, the administration of 1.0 mg ozanimod significantly reduced the loss of cortical grey matter and thalamic volume compared to interferon beta-1a [153]. Long-term safety—24 months—of the same doses of ozanimod was evaluated in an independent phase II clinical trial, RADIANCE (safety and efficacy of ozanimod versus interferon beta-1a in relapsing multiple sclerosis). Consistently, the safety profile of the compound was confirmed in the long term, and similar results on brain volume loss have been observed [154]. Despite the similarity with siponimod, the safer profile of this therapeutic agent made ozanimod suitable for further investigations. However, less is known about its activity in the CNS. Ozanimod treatment significantly reduced plasma levels of neurofilament light chain, a human biomarker for neurodegeneration, which positively correlates with spinal cord inflammation and demyelination in EAE and cuprizone mice [155]. In vivo, treatment with ozanimod showed reduced axonal breaks in the corpus callosum, and enhanced functional abilities in cuprizone-induced demyelination [156]. Besides, ozanimod directly modulates astrocyte activity in vitro via the activation of Akt and ERK pathways, thereby inhibiting the release of lipopolysaccharides (LPS)-induced pro-inflammatory cytokines, such as IL-1β, tumor necrosis factor α (TNFα), and IL-6 [156]. Ozanimod can cross the BBB, and the few pieces of evidence of its purpose at the CNS level let to think of a possible direct implication on oligodendrolineage cells, though its precise effect on OPCs and oligodendrocytes remains largely unknown thus far.

### 3.2. New Modulators in Ongoing Trials

Many other S1PR modulators are nowadays in different clinical trial phases for MS (Table 3). One of these compounds is ponesimod, a potent agonist of S1PR1 (Table 3) that displays 650 times higher selectivity compared to its endogenous ligand S1P [157] and is currently in the pre-registration phase for RRMS. The effect of ponesimod (30 mg/Kg) in EAE mice was assessed by prophylactic (after 1-day post-immunization (d.p.i)) and therapeutic (after 15 d.p.i) oral administration. Interestingly, both preventive and therapeutic administration leads to a significant reduction in the clinical score even at the chronic stage of the disease [158]. The positive outcomes obtained in the chronic phase, where the immunological component is less predominant, suggest ponesimod could have another mechanism of action besides the immunomodulatory one. It would be interesting to investigate if ponesimod could improve (re)myelination processes by directly intervening on oligodendroglia.

Ceralifimod is a potent agonist of S1P receptors, exhibiting a high affinity to S1PR1 and S1PR5 (see Table 3). The immunomodulatory effect of ceralifimod has been investigated in a clinical study conducted on healthy subjects. Ceralifimod treatment showed a dose-dependent decrease in the number of lymphocytes with a more pronounced depletion of CD19^+^, CD4^+^, and CD4^+^/CD25^+^ cells, despite no effect on NK cells number [160]. From preliminary studies, ceralifimod administration shows a reduced frequency of bradycardia and atrioventricular block compared to fingolimod [160]. The potential role of ceralifimod on the CNS has not yet been clarified; however, some in vivo electrophysiology experiments have been conducted on EAE rats. Administration of ceralifimod can recover the altered baseline visual and somatosensory evoked-potential, which are delayed in both acute and chronic stages of the disease [166]. Similar to the other novel compounds, there is no direct evidence that ceralifimod directly interacts with OPCs and oligodendrocytes.

Amiselimod is a second-generation S1PR modulator, and because of its immunomodulatory effects, it is now in phase II of clinical trials for RRMS. In vivo, it is rapidly converted into the active form (amiselimod-P), which shows a high affinity to S1PR1, lower to S1PR5, and minimal agonistic activity towards S1PR4. Even though the conversion of amiselimod into the active form is significantly lower compared to fingolimod, the benefit-risk profile appears to be safer. Indeed, amiselimod is the first of its category that does not show a decrease in heart rate after the first administration at the clinical dose in both rats and humans [162,167].

## 4. Conclusions

S1PR modulators have been developed in the last decades as DMTs to treat many inflammatory diseases, in particular, MS. Three of these compounds have already been approved by the FDA to treat MS, while many others are now in clinical testing. Most of these compounds are in ongoing trials for RRMS while few molecules are in clinical trials to treat SPMS, and until now, no one of them is under consideration for PPMS. While RRMS seems to be driven by inflammation, the chronic demyelination that occurs in SPMS and PPMS seems to not be affected by the available immunomodulators—rendering the discovery of drugs that can directly modulate demyelination/remyelination processes crucial. For this reason, investigating the effect of S1PR modulators on OPC proliferation and differentiation becomes intriguing and might revealing promising insights into when to target specific receptors. The current literature shows that oligodendroglial cells express specific S1PRs that are characteristic for distinct differentiation stages. S1PR1, mainly expressed by OPCs, decreases its expression during differentiation. In accordance, low levels of S1PR1 are detectable in oligodendrocytes, which predominantly express S1PR5. Starting from this evidence, it is plausible that S1PR1 activation could be involved in early OPC differentiation and migration, while S1PR5 activation might mediate differentiation in the later myelination stages. Partly linked to this is another essential aspect, which is the dose-dependent effect of S1PR modulators. Many in vitro studies show that low doses can exert more positive effects compared to higher concentrations. Although the underlying mechanism remains unclear, two main hypotheses can be taken into consideration. The first idea is related to the receptor affinity of the compound. Low concentrations of a compound can activate, with high selectivity, specific receptor subtypes. Differently, high concentrations of the ligand may non-specifically activate more receptors, losing the receptor subtype-specific activity. The second hypothesis is related to the antagonist-like mechanism. Low doses may promote S1PR internalization, which in turn may induce the transcription of other S1PRs, and therefore, subsequently increase their membrane expression. This resensitization could lead to prolonged responsiveness of the cells to endogenous and exogenous ligands.

The data collected from studies conducted with the first-generation modulators, even if inconclusive, are promising. Further investigations are required to understand the mechanism behind the crosstalk between S1PRs and remyelination. Therefore, it would be interesting to investigate the OPC/oligodendrocyte effect of newly developed S1PR modulators, while taking into account the considerations above, including timing and dosing of treatment. In addition, the higher receptor subtype affinity and safer benefit-risk profile can give the rationale to investigate further the potential neuroprotective role of these molecules and their direct impact on OPCs, oligodendrocytes, and more in general, on the remyelination processes.

## Figures and Tables

**Table 1:** Pro-myelinating compounds in ongoing clinical trials for multiple sclerosis (MS) treatment. Semaphorin-4D (SEMA4D); progressive MS (PMS); relapsing-remitting MS (RRMS); secondary progressive MS (SPMS); leucine-rich repeat.

Compound	Classification	Mechanism of Action	Indication	Clinical Trial	References
**Antisemaphorine 4D** (VX15/2503) Catalent Pharma Solutions^©^	Humanizes monoclonal antibody	anti-SEMA4D. Blocks the interaction between SEMA4D and its receptors	PMS; RRMS	Phase I	[57]
**Liothyronine** (L-T3 liothyronine sodium)	Nuclear Hormone Agonists	Thyroid receptor agonist	MS	Phase I	[58]
**rHIgM22** Acorda Therapeutics, Inc.^©^	Human IgM antibody	Not clarified	MS	Phase I	[59]
**Domperidone**	D2/D3 dopamine receptor antagonist	Increases prolactin serum levels	SPMS	Phase II	[60]
**Clemastine** (Clematine fumate meclastin)	Antihistamine	H1 antihistamine antagonist M1/M3 muscarinic receptors reverse antagonist	RRMS	Phase II	[61,62]
**GSK239512** GlaxoSmithKline^©^	Antihistamine	H3 receptor antagonist	RRMS	Phase II	[63]

**Table 2:** Sphingosine-1-phosphate receptor (S1PR) modulators approved for multiple sclerosis (MS) treatment. Features: receptor affinity, half-life (t1/2), time to reach the highest plasma concentration (Tmax), required phosphorylation (pro-drug), and indication for MS treatment (relapsing-remitting MS (RRMS); secondary progressive MS (SPMS) and clinically isolated syndrome (CIS)).

Compound	Receptor Affinity		T_1/2_ (Hours)	T_max_ (Hours)	Pro-Drug (Phosphorylation)	Indication	References
	High	Low					
**Fingolimod** (FTY720 Gilenya) Novartis^©^	S1PR1 (EC_50_ = 0.3 nM) S1PR3 (EC_50_ = 0.9 nM) S1PR5 (EC_50_ = 0.50 nM)	S1PR2 (EC_50_ > 10,000 nM) S1PR4 (EC_50_ = 345 nM)	144–216	12–16	+	RRMS	[99,100,101]
**Siponimod** (BAF312 Mayzent) Novartis^©^	S1PR1 (EC_50_ = 0.39 nM) S1PR5 (EC_50_ = 0.38 nM)	S1PR2 (EC_50_ > 10,000 nM) S1PR3 (EC_50_ > 1000 nM) S1PR4 (EC_50_ > 750 nM)	26–33	6–8	-	RRMS SPMS CIS	[102,103,104,105]
**Ozanimod** (RPC1063 Zeposia) Celgene^©^	S1PR1 (EC_50_ = 0.41 nM) S1PR5 (EC_50_ = 11 nM)	S1PR2 (EC_50_ > 10,000 nM) S1PR3 (EC_50_ > 10,000 nM) S1PR4 (EC_50_ > 7 nM)	15–17	8–12	-	RRMS SPMS CIS	[101,106]

**Table 3:** Sphingosine-1-phosphate receptor (S1PR) modulators in ongoing clinical trials for multiple sclerosis (MS) treatment. Features: receptor affinity, half-life (t1/2), time to reach the highest plasma concentration (Tmax), required phosphorylation, and indication for multiple sclerosis (MS) treatment (relapsing-remitting MS (RRMS)).

Compound	Receptor Affinity		T_1/2_ (Hours)	T_max_ (Hours)	Pro-Drug (Phosphorylation)	Indication	References
	High	Low					
**Ponesimod** (ACT-128800) Actelion^©^	S1PR1 (EC_50_ = 5.7 nM) S1PR5 (EC_50_ = 11 nM)	S1PR2 (EC_50_ > 10,000 nM) S1PR3 (EC_50_ > 10,000 nM) S1PR4 (EC_50_ > 7000 nM)	21–33	2–4	-	RRMS (phase III)	[159]
**Ceralifimod** (ONO-4641) Ono Pharmaceutical^©^	S1PR1 (EC_50_ = 0.273 nM) S1PR5 (EC_50_ = 0.334 nM)	S1PR2 (EC_50_ > 30,000 nM) S1PR3 (EC_50_ > 30,000 nM)	82–89	4–6	-	RRMS (phase II)	[160,161]
**Amiselimod** (MT-1303) Mitsubishi tanabe pharma corporation^©^	S1PR1 (EC_50_ = 0.075 nM) S1PR5 (EC_50_ = 0.47 nM) S1PR4 (EC_50_ = 122 nM)	S1PR2 (EC_50_ > 10,000 nM) S1PR3 (EC_50_ > 10,000 nM)	408	12–16	+	RRMS (phase II)	[162,163]
**GSK2018682** GlaxoSmithKline^©^	S1PR1 (EC_50_ = 0.07 nM) S1PR5 (EC_50_ = 0.072 nM)	S1PR3 (EC_50_ > 1000 nM)	48–63	4–9	-	RRMS (phase I)	[164]
**CS-0777** Daiichi Sankyo^©^	S1PR1 (EC_50_ = 1.1 nM) S1PR5 (EC_50_ = 21 nM)	S1PR3 (EC_50_ = 350 nM) S1PR4 (no data)	9–11	8–10	+	MS (phase I)	[165]

## Abbreviations

MS	Multiple sclerosis
CNS	Central nervous system
S1PR	Sphingosine-1-phosphate receptor
OPC	Oligodendrocyte progenitor cell
Shh	Sonic hedgehog
Olig	Oligodendrocyte transcription factor
Nkx6.1	Homeobox protein Nkx-6.1
Sox	Sex determining region Y-box
PDGFα	Platelet-derived growth factor-alpha
PDGFRα	PDGFα receptor
PLP	Protein proteolipid protein
MBP	Myelin basic protein
MAG	Myelin-associated glycoprotein
GalC	Galactocerebroside
MOG	Myelin-oligodendrocyte glycoprotein
BBB	Blood-brain barrier
ROS	Reactive oxygen species
DMTs	Disease modifying therapies
SEMA4D	Semaphorin-4D
PMS	Progressive multiple sclerosis
SPMS	Secondary progressive multiple sclerosis
S1P	Sphingosine-1-phosphate
SphK	Sphingosine kinase
MAP	Mitogen-activated protein
PI3K	Phosphoinositide 3-kinase
PLC	Phospholipase C
NK	Natural killer
ERK	Extracellular signal-regulated kinase
Akt	Protein kinase B
PIP3	Phosphatidylinositol (3,4,5)-trisphosphate
PDK1	3-phosphoinositide dependent protein kinase-1
CREB	cAMP response element-binding protein
p38MAPK	p38 mitogen-activated protein kinase
MEK	Mitogen-activated protein kinase
ROCK	Rho-associated protein kinase 1
CRMP	Collapsin response mediator protein
FDA	Food and drug administration
RRMS	Relapsing-remitting multiple sclerosis
EAE	Experimental autoimmune encephalomyelitis
CIS	Clinically isolated syndrome
BDNF	Brain-derived neurotrophic factor
LIF	Leukemia inhibitory factor
HBEGF	Heparin-binding EGF-like growth factor
LPC	Lysolecithin
Nrf2	Nuclear factor erythroid 2–related factor 2
HO-1	Heme oxygenase
NQO1	NAD(P)H: quinone acceptor oxidoreductases-1
p.o.	post-onset
PPMS	Primary progressive form of MS
d.p.i	Day post immunization
GLAST	Glutamate-aspartate transporter
GLT-1	Glutamate transporter-1
IL	Interleukin
ARR	Annualized relapse rate
LPS	Lipopolysaccharides
TNFα	Tumor necrosis factor α
